# Prevalence and cardiovascular associations of elevated lipoprotein(a) in people living with HIV: A cross-sectional study

**DOI:** 10.1016/j.clinme.2026.100625

**Published:** 2026-07-16

**Authors:** Marta Lago, Marta Salas, Ana Torres, Cristina Diez, Chiara Fanciulli, Leire Perez, Francisco Tejerina, Elena Bello, Jose Maria Bellon, Patricia Muñoz, Luis Antonio Álvarez-Sala, Irene González-Martinez, Teresa Aldámiz-Echevarria

**Affiliations:** a Servicio de Medicina Interna Gregorio Marañón General University Hospital, Madrid, Spain; b Hypertension and Vascular Risk Unit, Internal Medicine, Gregorio Marañón General University Hospital, Madrid, Spain; c Instituto de Investigación Sanitaria Gregorio Marañón (IiSGM), Madrid, Spain; d Servicio de Microbiología Clínica y Enfermedades Infecciosas, Gregorio Marañón General University Hospital, Madrid, Spain; e CIBERINFEC, ISCIII - CIBER de Enfermedades Infecciosas, Instituto de Salud Carlos III, Spain; f CIBER Enfermedades Respiratorias (CIBERES), Bunyola, Spain; g Servicio de Bioquimica, Gregorio Marañón General University Hospital, Madrid, Spain

**Keywords:** HIV, Lipoprotein(a), Cardiovascular risk, Residual risk

## Abstract

**Background:**

Lipoprotein(a) [Lp(a)] has emerged as an important cardiovascular risk factor. However, the prevalence and determinants of elevated Lp(a) in people living with HIV (PLHIV) remain insufficiently characterised. Since PLHIV have an increased cardiovascular risk even under effective virological control, our aim was to explore the prevalence of elevated Lp(a) and its association with cardiovascular risk in this population.

**Methods:**

We conducted a single-centre observational study in adult PLHIV with sustained virological suppression and without active oncological or infectious comorbidities. According to most international consensus statements, elevated Lp(a) was defined as levels >50 mg/dL.

**Results:**

A total of 186 PLHIV were included. Of these, 21% had Lp(a) levels >50 mg/dL. Individuals with elevated Lp(a) had significantly higher total cholesterol, Low-density lipoprotein cholesterol (LDL-C) and apolipoprotein B levels, as well as a higher odds of a cardiovascular event [odds ratio (OR) 5.3, p = 0.018]. Although the proportion of patients receiving lipid-lowering therapy was significantly higher among those with elevated Lp(a), the percentage achieving LDL-C targets according to cardiovascular risk was significantly lower compared with patients with Lp(a) <50 mg/dL. No association was observed between elevated Lp(a) and antiretroviral therapy regimens, CD4 count, CD4/CD8 ratio or duration of HIV infection.

**Conclusions:**

In our study, elevated Lp(a) was present among 21% of virologically suppressed PLHIV and was associated with an adverse lipid profile and cardiovascular events, whereas no association was observed with HIV-related factors. Lp(a) may represent a marker of residual cardiovascular risk in this population and could help refine cardiovascular risk stratification beyond traditional lipid parameters.

## Highlights

• Elevated lipoprotein(a) (>50 mg/dL) was present in 21% of virologically suppressed people living with HIV.
• High Lp(a) levels were associated with an adverse lipid profile, including higher LDL cholesterol and apolipoprotein B.
• Elevated Lp(a) was strongly associated with cardiovascular events, independently of HIV-related factors.
• Patients with high Lp(a) less frequently achieved LDL cholesterol targets despite more frequent statin use.
• Lp(a) may identify residual cardiovascular risk beyond traditional lipid parameters in people living with HIV.

## Introduction

Cardiovascular disease is the leading cause of mortality among people living with HIV (PLHIV). Although this is also true in the general population, PLHIV have an intrinsically increased cardiovascular risk related to HIV infection itself and the chronic inflammatory response it induces, independent of traditional atherosclerotic risk factors which are, mainly, hypertension, diabetes mellitus, smoking and dyslipidaemia. In addition, HIV-specific factors such as chronic immune activation, persistent inflammation, antiretroviral therapy exposure and a higher prevalence of metabolic abnormalities may further contribute to cardiovascular risk in this population. It is estimated that the relative risk of cardiovascular events is 1.5–2 times higher in PLHIV compared with non-infected individuals.^1^, ^2^, ^3^, ^4^ The REPRIEVE trial demonstrated that the use of pitavastatin in PLHIV, even among individuals with only moderate cardiovascular risk, reduces the incidence of ischaemic cardiovascular events.^5^, ^6^

Lipid abnormalities are among the main contributors to atherosclerotic cardiovascular disease (ASCVD). Low-density lipoprotein cholesterol (LDL-C) is not solely a risk biomarker, but a causal factor in the pathophysiology of ASCVD. A dose-dependent relationship exists between LDL-C exposure and ASCVD risk, with elevated LDL-C levels preceding disease onset. Furthermore, ASCVD risk depends not only on the current LDL-C concentration, but also on the cumulative duration of LDL-C exposure.^7^

However, LDL-C does not always accurately reflect the total burden of atherogenic lipoproteins. In clinical conditions such as diabetes mellitus, metabolic syndrome, insulin resistance and hypertriglyceridaemia, small dense LDL-C particles are more prevalent, and discordance between LDL-C and other atherogenic markers may occur. In these settings, non-high-density lipoprotein cholesterol (non-HDL-C) provides a broader measure of atherogenic cholesterol, encompassing LDL-C, triglyceride-rich lipoproteins (TRLs), remnant particles and lipoprotein(a) [Lp(a)], and may better predict ASCVD risk than LDL-C alone.^8^ Apolipoprotein B (apoB) reflects the total number of circulating atherogenic lipoprotein particles, as each LDL, VLDL, IDL, remnant particle and Lp(a) particle contains one apoB molecule. Consequently, apoB is considered a direct measure of atherogenic particle burden and may improve cardiovascular risk stratification, particularly in individuals with metabolic disorders or discordance between LDL-C and particle number.^9^

The negative impact of certain antiretroviral drugs on lipid profiles is well established.^10^, ^11^ In recent years, additional lipid-related biomarkers associated with cardiovascular risk have gained increasing attention. Among them, Lp(a) has emerged as a relevant predictor of atherosclerotic cardiovascular disease in the general population due to its atherogenic, prothrombotic and proinflammatory properties, adding to traditional cardiovascular risk factors.^12^ Although Lp(a) is structurally similar to LDL-C, it is characterised by the presence of apolipoprotein(a) [apo(a)], which confers a more pronounced atherothrombotic profile.^13^ Circulating Lp(a) concentrations are largely genetically determined and vary according to ethnicity.^14^, ^15^, ^16^ Levels of Lp(a) >50 mg/dL have been associated with an increased risk of coronary artery disease and cardiovascular mortality,^17^, ^18^ while levels >180 mg/dL confer a cardiovascular risk comparable to that of heterozygous familial hypercholesterolaemia.^19^, ^20^

Beyond genetic determinants, non-genetic factors may also contribute to elevated Lp(a) levels, including acute and chronic proinflammatory states.^21^ In PLHIV, cardiovascular risk is closely linked to persistent inflammation secondary to HIV infection, which may persist despite effective virological suppression. However, data regarding Lp(a) levels in PLHIV are limited, and much of the available evidence remains contradictory.

Given the limited and conflicting data on Lp(a) in PLHIV, we aimed to evaluate its prevalence and cardiovascular associations in a well-controlled HIV cohort.

## Methods

### Study design and population

We conducted a single-centre observational study in PLHIV who were followed at Hospital General Universitario Gregorio Marañón, a tertiary-care centre located in Madrid, Spain, where approximately 3,300 individuals with HIV receive regular clinical care.

Participants were recruited sequentially among patients attending routine follow-up laboratory testing between 15 May and 15 June 2023, and between 1 June and 15 July 2024. Two recruitment periods were used in order to achieve the estimated sample size after an initial exploratory analysis of the prevalence of Lp(a) levels >50 mg/dL. All participants provided written or oral informed consent prior to inclusion. None of the patients approached for participation declined to take part in the study.

Inclusion criteria were: age ≥18 years; stable antiretroviral therapy for at least 3 months prior to inclusion; sustained virological suppression during the same period; absence of active oncological disease or active or recent infection (within the previous 15 days); and no hospital admission in the 15 days prior to inclusion. Pregnant women were excluded.

Race/ethnicity was recorded at enrolment according to self-identification as a single categorical variable (White, Hispanic/Latino, Black or Asian).

### Clinical and laboratory assessment

In addition to routine laboratory parameters obtained as part of standard HIV follow-up, all participants underwent an extended lipid profile assessment, including measurement of Lp(a) and apoB taken simultaneously.

Lp(a) mass concentrations were measured using a particle-enhanced immunoturbidimetric assay performed on the Alinity c system (Abbott Laboratories, Illinois, USA). ApoB concentrations were measured by immunonephelometry using a Siemens BNII nephelometric analyser (Siemens Healthineers, Erlangen, Germany). All analyses were performed in the hospital’s central clinical laboratory according to the manufacturers’ instructions.

ASCVD was defined as coronary heart disease, cerebrovascular disease and peripheral artery disease. These events were confirmed by medical records after HIV diagnosis.

SCORE 2 cardiovascular risk scale was calculated at study inclusion.

Elevated Lp(a) levels were defined as values >50 mg/dL (equivalent to approximately 125 nmol/L), in accordance with most international scientific society recommendations, which consider this threshold clinically significant due to its association with increased risk of atherosclerotic vascular disease, particularly coronary artery disease, and cardiovascular mortality.^19^ Target values for LDL-C, non-HDL-C and apoB were defined according to the most recent European Society of Cardiology guidelines.^22^

For comparative analyses, participants were stratified according to Lp(a) concentration into two groups: elevated Lp(a) (>50 mg/dL) and non-elevated Lp(a) (≤50 mg/dL).

### Ethical approval

The study was approved by the Research Ethics Committee of Hospital General Universitario Gregorio Marañón (approval number 15/2023) and was conducted in strict accordance with established ethical standards and regulatory requirements.

### Statistical analysis

The sample size was determined to estimate the prevalence of elevated Lp(a) concentrations [Lp(a) >50 mg/dL], which has been reported to be approximately 20% in the general population.^19^ Assuming a 95% confidence level and a precision of ±6% points, a minimum of 171 participants was required. To account for potential missing data or non-evaluable measurements, the sample size was increased by approximately 9%, yielding a final cohort of 186 individuals.

This sample size also provides 90% power, with a two-sided alpha of 0.05, to detect between-group differences corresponding to a standardised mean difference of 0.6 or greater. According to Cohen’s classification,^23^ an effect size of 0.5 is considered to be of ʻmoderate’ magnitude.

Normality was assessed using the Kolmogorov–Smirnov test. Differences between the elevated Lp(a) group (>50 mg/dL) and the non-elevated Lp(a) group (≤50 mg/dL) were evaluated using parametric tests (Student’s *t*-test) or non-parametric tests (Mann–Whitney *U* test), as appropriate. Categorical variables were compared using the chi-square (χ²) test or Fisher’s exact test, as appropriate.

The association between continuous Lp(a) and apoB concentrations was evaluated using Spearman’s rank correlation coefficient.

A multivariable logistic regression analysis was performed to evaluate factors associated with Lp(a) levels >50 mg/dL. Variables were selected *a priori* based on clinical relevance (age, sex and ethnicity) and on statistically significant associations observed in bivariate analyses (LDL-C >150 mg/dL). Given the limited number of events, a parsimonious modelling strategy was adopted to minimise overfitting and ensure an adequate proportion of events per variable. The model’s discriminatory capacity was assessed using the area under the receiver operating characteristic (ROC) curve (AUC), with its corresponding 95% confidence intervals. Calibration was assessed using graphical tests and the Hosmer–Lemeshow test. Multivariable analyses were performed using full case analyses, including only patients with available data for all variables in the model. Missing data were minimal and analyses were performed using complete-case data. No missing values were present for variables included in the multivariable model. The Variance Inflation Factor (VIF) study showed no collinearity in the independent variables.

Statistical analyses were performed using Stata version 19 and IBM SPSS Statistics for Windows, version 25.0 (IBM Corp., Armonk, NY, USA). A p-value <0.05 was considered statistically significant.

## Results

A total of 186 patients were included. Of these, 39 patients (21%) had Lp(a) levels >50 mg/dL. Baseline characteristics stratified by Lp(a) status are shown in Table 1.

**Table 1:** Baseline characteristics according to Lp(a) levels.

Variable	Measure	Elevated Lp(a) (≥50 mg/dL) n = 39	Non-elevated Lp(a) (<50 mg/dL) n = 147	p-value
Age, years	*Median (IQR)*	52 (36–59)	47 (34–57)	0.418
**Sex/**g**ender**				
Male	*n (%)*	27 (69)	106 (72)	0.736
Cisgender female	*n (%)*	10 (26)	36 (25)	
Transgender female	*n (%)*	2 (5)	5 (3)	
**Race/**e**thnicity**				
White	*n (%)*	23 (59)	84 (57)	0.761
Hispanic/Latino	*n (%)*	14 (36)	58 (40)	
Black	*n (%)*	1 (2.6)	2 (1.4)	
Asian	*n (%)*	1 (2.6)	2 (1.4)	
**HIV transmission route**				
MSM	*n (%)*	20 (51)	80 (55)	0.523
IDU	*n (%)*	7 (18)	19 (13)	
Heterosexual	*n (%)*	0 (0)	7 (5)	
Unknown	*n (%)*	12 (31)	40 (27)	
**Cardiovascular risk factors**				
Hypertension	*n (%)*	9 (24)	34 (23)	1.000
Current smoker	*n (%)*	14 (36)	51 (35)	0.935
Former smoker	*n (%)*	5 (13)	17 (12)	
Diabetes mellitus	*n (%)*	1 (3)	18 (12)	0.132
**Cardiovascular risk (SCORE2)**				
Low	*n (%)*	25 (64)	106 (72)	0.095
Moderate	*n (%)*	9 (23)	25 (17)	
High	*n (%)*	0 (0)	9 (6)	
Very high	*n (%)*	5 (13)	7 (5)	
**History of ASCVD**				
Coronary artery disease	*n (%)*	5 (13)	2 (1.4)	**0.005**
Cerebrovascular disease	*n (%)*	1 (3)	1 (0.7)	0.376
Peripheral arterial disease	*n (%)*	1 (3)	1 (0.7)	0.376
**Lipid profile**				
Total cholesterol, mg/dL	*Median (IQR)*	181 (160–200)	173 (142–195)	**0.037**
LDL cholesterol, mg/dL	*Median (IQR)*	109 (93–135)	96 (75–116)	**0.003**
Apolipoprotein B, mg/dL	*Median (IQR)*	96 (83–122)	91 (72–106)	**0.014**
Triglyceride/HDL ratio	*Median (IQR)*	2.3 (1.9–3.3)	2.8 (1.8–4.0)	0.896
Lipid-lowering therapy	*n (%)*	19 (49)	38 (26)	**0.010**
LDL target achieved	*n (%)*	18 (46)	101 (69)	**0.014**
**HIV-related characteristics**				
CD4 count, cells/mm³	*Median (IQR)*	758 (489–1,043)	780 (551–1,122)	0.496
CD4/CD8 ratio	*Median (IQR)*	0.69 (0.43–1.16)	0.83 (0.60–1.12)	0.147
Previous AIDS-defining condition (CDC category C)	*n (%)*	14 (48)	36 (32)	0.131
Duration of HIV infection, years	*Median (IQR)*	12 (9–24)	14 (6–25)	0.992
**Current antiretroviral therapy**				
INSTI-based regimen	*n (%)*	32 (82)	113 (77)	0.664
NNRTI-based regimen	*n (%)*	5 (13)	30 (20)	0.397
PI-based regimen	*n (%)*	3 (8)	10 (7)	0.737
Dual therapy	*n (%)*	18 (46)	67 (46)	1.000
**Hepatitis C virus coinfection (past)**	*n (%)*	8 (21)	32 (22)	1.000

133 (72%) of the overall participants were men, 46 were cisgender women (24%) and seven were transgender women (4%). The median age was 49 years (IQR 35–58). Regarding ethnicity, 72 (39%) were Hispanic/Latino and the rest were not Hispanic/Latino, of whom 107 were White (57%), three Black (2%) and four Asian (2%). The median duration of HIV infection was 13.4 years (IQR 6.6–24.6). HIV transmission occurred through homosexual contact in 100 individuals (54%) and through injecting drug use in 26 (14%). Median CD4 cell count at the time of the study was 771 cells/mm^3^ (IQR 534–1,108), and the median CD4/CD8 ratio was 0.82 (IQR 0.58–1.15). Fifty patients (36%) had a prior AIDS-defining illness, and 40 (22%) had a history of hepatitis C virus coinfection that had been eradicated either spontaneously or following antiviral treatment.

As combination antiretroviral therapy is standard of care for HIV infection, all participants were receiving more than one antiretroviral drug, with a median of two drugs per regimen (range 2–3). The most common combination was lamivudine plus dolutegravir, an integrase strand transfer inhibitor (dual therapy). Lamivudine, given its near-universal use and lack of a recognised impact on lipid metabolism, was not analysed separately. Most patients (78%) were receiving an integrase strand transfer inhibitor-based antiretroviral regimen, while only 7.8% were receiving protease inhibitors. Regarding nucleoside reverse transcriptase inhibitors, 30.1% were treated with abacavir/lamivudine, 9.6% with tenofovir disoproxil fumarate (TDF) and 5.9% with tenofovir alafenamide (TAF).

Among the study population, 43 patients (23%) had hypertension and 19 (10%) had diabetes mellitus. The median body mass index (BMI) was 25 kg/m² (IQR 23–28). Sixty-five patients (35%) were current smokers and 22 (12%) were former smokers. Median total cholesterol was 175 mg/dL (IQR 147–196), with 34 patients (18%) presenting levels above 200 mg/dL. Median LDL-C was 99 mg/dL (IQR 78–119), and 10 patients (5%) had LDL-C levels >150 mg/dL. Median apoB level was 93 mg/dL (IQR 74–106). Median triglyceride levels were 113 mg/dL (IQR 91–161), median HDL-C was 45 mg/dL (IQR 38–54), and the median triglyceride-to-HDL ratio was 2.6 (IQR 1.85–3.8).

According to the SCORE2 cardiovascular risk scale, 131 patients (70%) had low cardiovascular risk, 34 (18%) moderate risk, nine (5%) high risk and 12 (7%) very high risk. Overall, 57 patients (31%) were receiving statins; of these, 21 had low cardiovascular risk, 20 had moderate risk, five had high risk and 11 had very high risk.

**Fig. 1:**
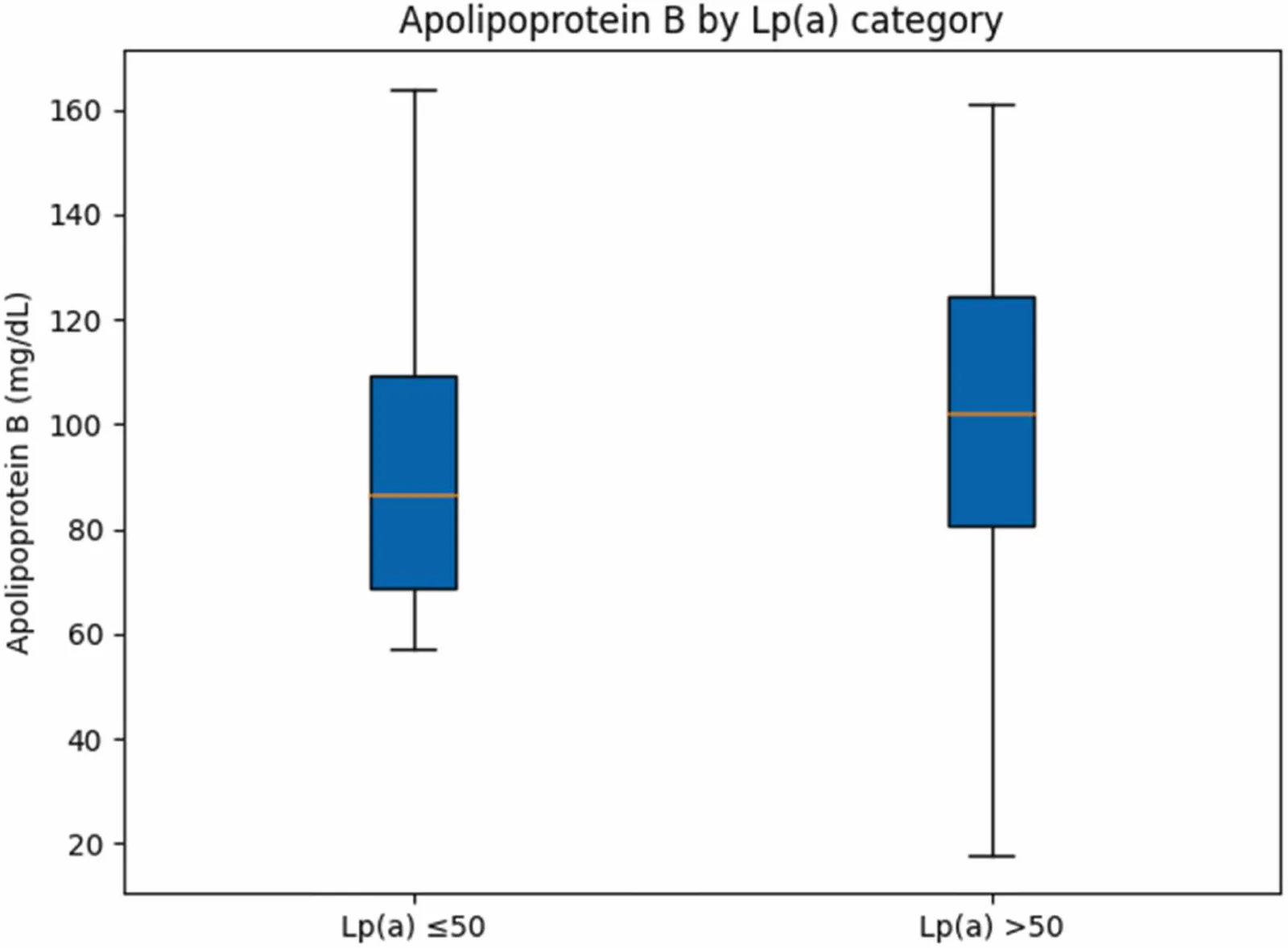
Association between Lp(a) and apoB levels. Distribution of apolipoprotein B (apoB) levels according to lipoprotein(a) [Lp(a)] category. Patients with Lp(a) >50 mg/dL showed significantly higher apoB concentrations compared with those with Lp(a) <50 mg/dL. (Spearman’s ρ = 0.12, p = 0.026).

Among patients with elevated Lp(a), 48.1% were receiving statin therapy, a significantly higher proportion than among those with Lp(a) <50 mg/dL (p = 0.010). Despite this, patients with Lp(a) >50 mg/dL had significantly higher median levels of total cholesterol (181 [IQR 160–200] vs 173 [IQR 142–195]; p = 0.037), LDL-C (109 [IQR 93–135] vs 96 [IQR 75–116]; p = 0.003), and apoB (96 [IQR 83–122] vs 91 [IQR 72–106], p = 0.014). Fig. 1 illustrates the association between Lp(a) and apolipoprotein B concentrations, showing a weak but statistically significant positive correlation (Spearman’s ρ = 0.12, p = 0.026).

Only 46.5% of patients with Lp(a) >50 mg/dL achieved LDL-C targets (Table 1 of Supplementary material) according to their cardiovascular risk category by SCORE-2, compared with 68.7% of patients with Lp(a) <50 mg/dL (p = 0.035).

Despite the limited number of ASCVD events observed, a significant association was found between Lp(a) levels >50 mg/dL and cardiovascular disease, supporting the role of Lp(a) as a marker of cardiovascular risk. To further characterise this association, a logistic regression analysis was performed. Elevated Lp(a) was associated with significantly higher odds of ASCVD (OR 5.3, p = 0.018; 95% CI 1.3–20.7).

Only two patients had Lp(a) levels >150 mg/dL. One was an 18-year-old Caucasian man with an Lp(a) level of 189 mg/dL, low cardiovascular risk, no additional traditional cardiovascular risk factors and optimal immunovirological control (CD4 count 1,216 cells/mm^3^, CD4/CD8 ratio 1.24), receiving lamivudine/dolutegravir. The second was a 54-year-old Caucasian man with an Lp(a) level of 162 mg/dL, active smoking, hypercholesterolaemia and chronic kidney disease, corresponding to moderate cardiovascular risk. He was receiving lipid-lowering therapy and had achieved LDL-C targets for his risk category. His HIV infection was also well controlled (CD4 count 1,516 cells/mm^3^, CD4/CD8 ratio 1.19) and he was receiving efavirenz and abacavir/lamivudine.

**Fig. 2:**
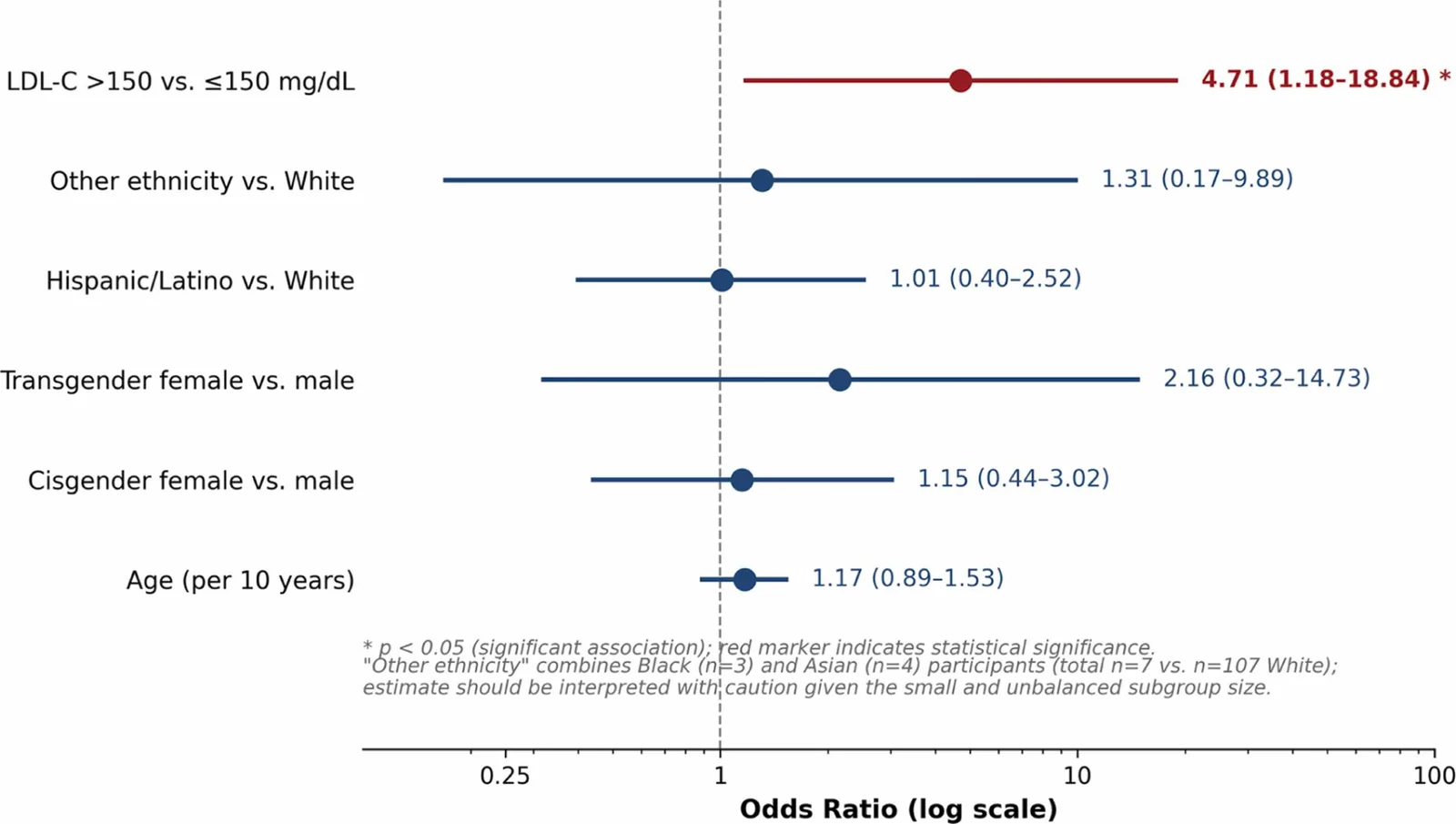
Factors associated with elevated lipoprotein(a).

A multivariable logistic regression analysis was performed to assess factors associated with Lp(a) levels >50 mg/dL. Covariates for the multivariable model were selected based on their clinical relevance (age, sex, ethnicity) or their statistically significant association with elevated Lp(a) in bivariate analyses (LDL-C >150 mg/dL). Other clinically relevant variables, including smoking status, hypertension, diabetes mellitus, BMI, statin use and antiretroviral therapy regimen, were not significantly associated with elevated Lp(a) in bivariate analyses (Table 1) and were therefore not included in the final model, in order to avoid overfitting given the limited number of cardiovascular events. No missing data were present for the variables included in the multivariable model. Only LDL-C levels >150 mg/dL were significantly associated with elevated Lp(a) (OR 4.7, p = 0.028; 95% CI 1.18–18.4), as shown in Fig. 2.

## Discussion

In this study conducted in PLHIV with adequate immunovirological control, the prevalence of elevated Lp(a) >50 mg/dL was 21%, a proportion similar to that reported in the general population.^24^ Elevated Lp(a) was associated with a more atherogenic lipid profile and a higher odds of cardiovascular events, whereas no association was observed with HIV-related characteristics, including CD4 cell count, CD4/CD8 ratio, duration of HIV infection or antiretroviral therapy exposure.

Due to its substantial impact on cardiovascular risk, Lp(a) has become a topic of growing interest, yet it remains poorly explored in PLHIV, and the available evidence is often contradictory. It is well established that Lp(a) concentrations are largely determined by apo(a) alleles, defined by the number of kringle IV repeats. Smaller apo(a) isoforms are associated with higher Lp(a) levels and increased cardiovascular risk. Studies in HIV-infected populations have linked elevated Lp(a) levels to specific apo(a) alleles that are more prevalent in individuals of African ancestry compared with Caucasians,^25^ as well as to higher CD4 lymphocyte counts, suggesting a relationship between elevated Lp(a) and increased inflammatory activity. In parallel, paediatric studies have reported higher Lp(a) levels in children living with HIV compared with age-matched uninfected controls, with levels converging during adolescence.^26^, ^27^

The potential effect of antiretroviral therapy (ART) on Lp(a) levels has also been explored, yielding conflicting results. In the SCRIPT study conducted in PLHIV in Ghana, individuals receiving ART had higher Lp(a) levels than those who were untreated.^28^ Conversely, a study from the Women’s Interagency HIV Study (WIHS) cohort comparing women with and without HIV reported that initiation of ART influenced apo(a) allele expression, resulting in a reduction of Lp(a) levels after treatment initiation.^29^ These discrepancies likely reflect differences not only in ART, but also in ethnicity, immune status and study design. In our cohort, predominantly composed of Caucasian and Latino individuals, we did not observe a higher prevalence of elevated Lp(a) compared with the general population, reinforcing the predominant role of genetic determinants over HIV-related factors in shaping Lp(a) levels.

Despite the absence of an increased prevalence, elevated Lp(a) in our cohort was strongly associated with cardiovascular events, with a more than fivefold increase in odds. Although the number of events was limited, the magnitude of this association is consistent with observations in the general population^30^, ^31^ and supports the role of Lp(a) as a potential clinically relevant marker of cardiovascular risk also in PLHIV. Importantly, this association was independent of traditional HIV-related parameters, underscoring the potential contribution of nontraditional mechanisms to residual cardiovascular risk in this population.

A particularly relevant finding of our study is the association between elevated Lp(a) levels and a more atherogenic lipid profile, characterised by significantly higher concentrations of total cholesterol, LDL-C, and apoB. ApoB reflects the total burden of atherogenic lipoprotein particles, including LDL-C, very-low-density lipoprotein remnants, and Lp(a), and is increasingly recognised as a more accurate marker of cardiovascular risk than LDL-C alone.^8^, ^9^ The coexistence of elevated Lp(a) and higher apoB levels suggests a substantially increased atherogenic load in these patients. Accordingly, apoB may represent a mechanistic link between elevated Lp(a) and cardiovascular events.

Importantly, patients with elevated Lp(a) were more frequently treated with statins yet were less likely to achieve lipid targets and experienced a higher burden of cardiovascular events, highlighting Lp(a) as a contributor to residual cardiovascular risk, not adequately addressed by standard lipid-lowering therapy. These findings reinforce the concept of persistent cardiovascular risk in PLHIV despite optimal virological control and lipid-lowering therapy, which appears to be largely driven by chronic inflammation.^32^, ^33^, ^34^ In this context, Lp(a) may act as an integrative marker of genetically determined risk and inflammation-driven vascular vulnerability, contributing to the excess cardiovascular risk observed in well-controlled PLHIV.

Our results are consistent with those reported by Kikuchi *et al*.^35^ Although that study found higher Lp(a) levels in PLHIV compared with controls, it is important to note that their population included a substantial proportion of African American individuals, whose genetic background differs markedly from that of our cohort. The authors proposed a link between elevated Lp(a) and coronary endothelial inflammatory dysfunction in PLHIV, a mechanism that has been identified as a key contributor to residual cardiovascular risk in HIV infection.^36^, ^37^, ^38^

Our findings suggest a similar paradigm in our population, namely the persistence of inflammation-driven residual cardiovascular risk in well-controlled PLHIV, with Lp(a) acting as a marker of this risk. Lp(a) identifies a subgroup of well-controlled PLHIV with residual cardiovascular risk that is not captured by traditional lipid parameters or current cardiovascular risk stratification tools. In this context, targeted treatment of elevated Lp(a), when available, could represent an effective therapeutic strategy to address residual cardiovascular risk in this population.

Our study has several limitations. Its single-centre and cross-sectional design precludes causal inference and may limit generalisability. Also, race/ethnicity was collected as a single self-reported variable, which precluded disaggregating Hispanic/Latino participants by race. The number of cardiovascular events was relatively small, and inflammatory biomarkers or imaging-based assessments of subclinical atherosclerosis were not available. Nevertheless, the sample size was adequate to estimate the prevalence of elevated Lp(a) and to explore its clinical associations in a well-characterised cohort of virologically suppressed PLHIV.

Given its clear association with residual cardiovascular risk, we believe that Lp(a) has substantial value in cardiovascular risk assessment and stratification in PLHIV. Our findings support current recommendations to measure Lp(a) at least once during adulthood, including in PLHIV.^39^ Incorporating Lp(a) into cardiovascular risk assessment may represent a simple yet meaningful step towards more personalised cardiovascular prevention in people living with HIV.

## Conclusions

In our study, the proportion of people living with HIV with Lp(a) levels >50 mg/dL was 21%, a prevalence similar to that reported in the general population. Participants with elevated Lp(a) had higher total cholesterol, LDL-C and apoB concentrations and a higher odds of cardiovascular events than those with lower Lp(a) levels. No differences were observed according to HIV-related characteristics, including antiretroviral therapy exposure, CD4 cell count, CD4/CD8 ratio or duration of HIV infection.

These findings suggest that Lp(a) may contribute to residual cardiovascular risk in PLHIV and may provide additional information beyond conventional lipid parameters for cardiovascular risk assessment. Larger prospective studies are needed to confirm its prognostic value and potential clinical utility in this population.

Measurement of Lp(a) may help refine cardiovascular risk stratification beyond traditional lipid parameters. Incorporating Lp(a) assessment into routine clinical practice could facilitate the identification of subgroups of PLHIV at higher cardiovascular risk and support the optimisation of preventive strategies, particularly in the context of emerging therapies specifically targeting Lp(a).

## CRediT authorship contribution statement

**Cristina Diez:** Writing – review & editing, Methodology. **Ana Torres:** Writing – review & editing, Methodology. **Marta Salas:** Writing – original draft, Investigation, Data curation. **Irene González-Martinez:** Investigation. **Marta Lago:** Writing – original draft, Investigation, Data curation. **Elena Bello:** Writing – review & editing. **Francisco Tejerina:** Writing – review & editing. **Leire Pérez:** Writing – review & editing. **Chiara Fanciulli:** Writing – review & editing. **Teresa Aldámiz-Echevarría:** Writing – original draft, Visualization, Validation, Supervision, Methodology, Investigation, Formal analysis, Data curation, Conceptualization. **Patricia Muñoz:** Supervision. **José María Bellón:** Formal analysis. **Luis Álvarez-Sala:** Supervision.

## Ethics approval and consent to participate

The study was approved by the Research Ethics Committee of Hospital General Universitario Gregorio Marañón (approval number 15/2023). All participants provided written or verbal informed consent prior to inclusion; as approved by the Research Ethics Committee. For participants whose follow-up visits were conducted by telephone rather than in person, verbal informed consent was obtained and documented in their electronic medical records. The study protocol, as approved by the Research Ethics Committee of Hospital General Universitario Gregorio Marañón, explicitly allowed verbal consent to be obtained from patients followed by telephone, provided it was documented in the medical record.

## Funding

This research did not receive any specific grant from funding agencies in the public, commercial, or not-for-profit sectors.

## Declaration of generative AI and AI-assisted technologies in the writing process

During the preparation of this work the authors used ChatGPT in order to translate into English. After using this tool/service, the authors reviewed and edited the content as needed and take full responsibility for the content of the published article.

## Declaration of competing interest

The authors declare that they have no known competing financial interests or personal relationships that could have appeared to influence the work reported in this paper.

## Data Availability

The datasets generated and/or analysed during the current study are available from the corresponding author on reasonable request.
