# Supplementary material for: Prevalence and cardiovascular associations of elevated lipoprotein(a) in people living with HIV: A cross-sectional study
Source: Clin Med (Lond). 2026 Jul 16;26(5):100625. doi: 10.1016/j.clinme.2026.100625 (PMC13499187; doi:10.1016/j.clinme.2026.100625)
Supplement: Supplementary file 1 [file mmc1.docx]

**SUPPLEMENTARY MATERIAL:**

Table: Reference thresholds for cardiovascular biomarkers evaluated in this study

|  | **NORMAL VALUES** |
| --- | --- |
| Lp(a) | < 50 mg/dL |
| LDL-C   - Low cardiovascular risk - Medium cardiovascular risk - High cardiovascular risk - Very high risk | <116 mg/dL  <100 mg/dL  <70 mg/dL  <55 mg/dL |
| non-HDL-C   - Low cardiovascular risk - Medium cardiovascular risk - High cardiovascular risk - Very high risk | <145 mg/dL  <130 mg/dL  <100 mg/dL  <85 mg/dL |
| apoB   - Low cardiovascular risk - High cardiovascular risk - Very high risk | <90 mg/dL  <80 mg/dL  <65 mg/dL |

Thresholds are based on current ESC/EAS lipid management guidelines (22). For Lp(a), concentrations ≥50 mg/dL are generally considered elevated and associated with increased ASCVD risk (19).
